# Influence of Ether-Functionalized Pyrrolidinium Ionic Liquids on Properties and Li^+^ Cation Solvation in Solvate Ionic Liquids

**DOI:** 10.1021/acs.jpcc.5c01403

**Published:** 2025-06-10

**Authors:** Michael J. Keating, Elijah Bernard, Martina Hove, Ho Martin Yuen, Mehreen Mughal, Surabh S. KT, James F. Wishart, Sharon Lall-Ramnarine, Robert J. Messinger, Elizabeth J. Biddinger

**Affiliations:** † Department of Chemical Engineering, 226673 The City College of New York, CUNY, New York, New York 10031, United States; ‡ Ph.D. Program in Chemistry, The Graduate Center of City University of New York, CUNY, New York, New York 10016, United States; § Department of Chemistry, 14782 Queensborough Community College of The City University of New York, Bayside, New York 11364, United States; ∥ 8099 Chemistry Division, Brookhaven National Laboratory, Upton, New York 11973, United States

## Abstract

Ionic liquids are tunable solvents composed entirely of ions that have properties desirable as electrolytes for lithium batteries such as nonflammability and a large electrochemical stability window. Solvate ionic liquids are a subclass of ionic liquids that consist of a glyme-based solvent and lithium salt in an equimolar ratio, where Li^+^ cation-glyme solvation interactions result in ionic liquid-like properties. LiG4TFSI is a well-studied solvate ionic liquid consisting of equimolar amounts of lithium bis­(trifluoromethylsulfonyl)­imide (LiTFSI) and tetraglyme (G4). In this work, pyrrolidinium ionic liquids with ether-functionalized side chains were synthesized, containing either one ether (EO1) moiety or three ether (EO3) moieties and mixed with LiG4TFSI to form a new class of electrolyte mixtures. Their physical and transport properties, as well as ion solvation structures, were characterized by electrochemical, thermal, rheological, and spectroscopic measurements. The conductivity of the electrolyte mixture composed of EO1:LiTFSI:G4 in a 1:1:1 molar ratio is 2.54 mS/cm at 30 °C, compared to 1.53 mS/cm for LiG4TFSI, an increase of 67%. A significant decrease in the conductivity to 0.279 mS/cm is observed for the EO3:LiTFSI:G4 mixture in a 1:1:0.4 molar ratio. Pulsed-field gradient nuclear magnetic resonance (PFG-NMR) measurements revealed that the EO1 cation diffuses significantly faster than the EO3 cation in their respective mixtures. Liquid-state ^13^C NMR experiments indicate that Li^+^ cations preferentially coordinate with tetraglyme. Li^+^ cations do not coordinate with the EO1 cation and coordinate with the EO3 ether side chains only at lower concentrations of tetraglyme. We hypothesize that the oligoether EO3 cation competes with G4 and TFSI^–^ for lithium cation solvation in G4-deficient compositions, leading to a largely adverse effect on the mass transport properties of the electrolyte.

## Graphical abstract

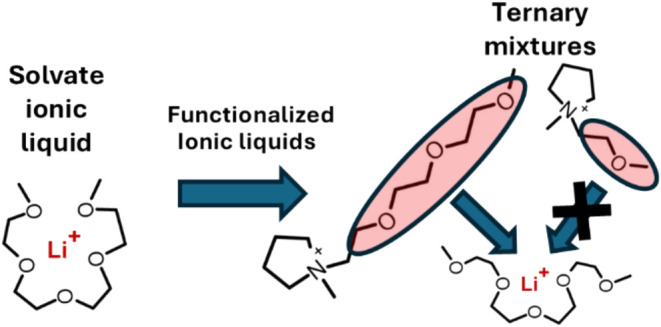

## Introduction

Lithium metal batteries (LMB) are candidates for next-generation electrochemical energy storage due to their high energy densities; however, they have issues with specific capacity retention and greater flammability concerns when paired with organic-based electrolytes.^1,2^ An approach to improving them is designing safer electrolytes that facilitate stable battery operation. Ionic liquids (ILs)^3,4^ and solvate ionic liquids (SILs)^5^ are being explored as materials for Li-based battery electrolytes due to their negligible vapor pressure and low flammability, as well as their large electrochemical stability window, typically between 5 V and 6 V.^6,7^ ILs are salts that melt below 100 °C and consist completely of cations and anions.^8^ ILs are tunable “designer” solvents due to the numerous possible cation and anion combinations as well as the ability to modify functionality. SILs are a subclass of ionic liquids made by mixing high concentrations of certain salts, usually lithium salts, with short-chain polyether molecules called glymes.^9^ LiG4TFSI is a SIL composed of equimolar amounts of tetraethylene glycol dimethyl ether (tetraglyme, G4) and lithium bis­(trifluoromethylsulfonyl)­imide. Since tetraglyme has 5 ether oxygens, LiG4TFSI has an [O]/[Li^+^] ratio = 5. SILs will have an [O]/[Li^+^] ratio close to the solvation number of Li^+^ to help mitigate the oxidation of the ether functionality by glyme molecules coordinating to Li^+^.^6^

As battery electrolytes, ILs and SILs are limited by high viscosities, leading to poor ion mass transport.^10^ Another shortcoming of common ILs is that their melting points are often near ambient temperature, which limits the scope of their applications. The addition of higher concentrations of lithium salts to ILs and SILs can further reduce ion transport and the liquid temperature window. However, at higher salt concentrations, researchers have observed modes of ion conduction other than conventional vehicular conduction, notably ion-hopping conduction, where the ion transports faster than the solvent.^11–16^

The Li^+^ solvation structure has a profound impact on the properties of the electrolyte and battery functionality. Kautz et al. also demonstrated solvation environments can be utilized to improve charge transfer kinetics at the electrode interface, allowing for faster charging of Li-ion batteries.^17^ Low temperature performance of lithium metal batteries has been improved by using weakly coordinating solvents to modify the coordination environment.^18,19^ Understanding the solvation of Li^+^ helps explain much of the transport^20,21^ and electrochemical^22,23^ properties of the electrolyte. The solvation environments of Li^+^ cations also have significant effects on regulating solid electrolyte interface (SEI) formation.^24–26^ Changing the functionality of ILs is often used to modify physical properties^27–30^ and the liquid structure characteristics^31,32^ of the electrolyte. Specifically, ether-functionalized ILs as tailorable solvents have been studied with lithium and other metal cations for battery applications.^33–35^ Previously, IL-glyme electrolytes have been studied in lithium–sulfur,^36–39^ lithium–oxygen,^40–44^ and lithium–LiFePO_4_ ^45^ battery systems and demonstrated improved conductivity through a wide temperature range.

In this work, functionalized ILs were synthesized and mixed with the solvate ionic liquid LiG4TFSI to understand how the physical properties, mass transport properties, and Li^+^ solvation structure can be tuned in the ternary mixtures. The addition of functionalized ILs to LiG4TFSI added a component to compete with G4 and TFSI^–^ in the Li^+^ primary solvation shell. We investigated mixtures of tetraglyme and LiTFSI and introduced two pyrrolidinium-based ILs with side chains containing one ether group (*N*-(2-methoxyethyl)-*N*-methyl pyrrolidinium bis­(trifluoromethylsulfonyl)­imide, [EOM mPyrr]­[TFSI], EO1) and three ether groups (*N*-methyl-*N*-(2-(2-(2-methoxyethoxy)­ethoxy)­ethyl) pyrrolidinium bis­(trifluoromethylsulfonyl)­imide, [(EO)_3_M mPyrr]­[TFSI], EO3). These ILs were selected because ether-functionalized pyrrolidinium ILs typically have a lower viscosity than their alkyl-side chain analogues due to the increased flexibility and curling of the ether-functionalized side chain.^29,46,47^ Additionally, longer ether side chains have been shown to coordinate Li^+^.^20,33,46^ Adding the EO1 and EO3 ILs in a 1:1:1 IL:LiTFSI:G4 molar amount increased the [O]/[Li^+^] ratio from 5 in LiG4TFSI to 6 and 8, respectively. To better understand how the addition of the ILs influences the electrolyte, compositions with equimolar amounts of IL and LiTFSI but less glyme were mixed to maintain [O]/[Li^+^] to 5. By varying the chain length, we sought to further investigate the effect of the ether side chains on the Li­[G4]^+^ cation complex. The structures and compositions studied are summarized in Table 1 and Figure 1.

**Figure 1:**
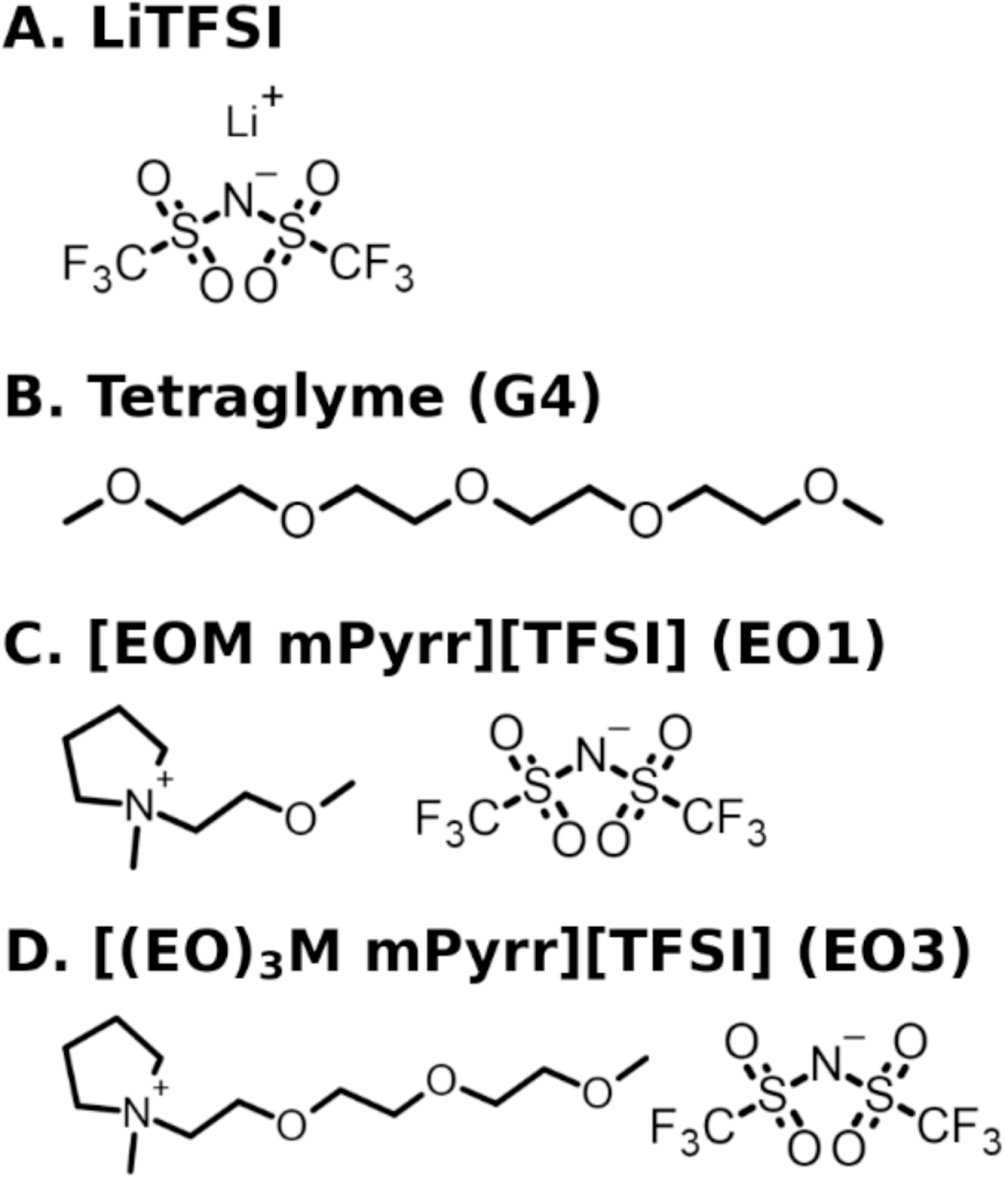
Chemical structures for (A) lithium bis­(trifluoromethylsulfonyl)­imide (LiTFSI), (B) tetraethylene glycol dimethyl ether (tetraglyme, G4), (C) (*N*-(2-methoxyethyl)-*N*-methyl pyrrolidinium bis­(trifluoromethylsulfonyl)­imide, [EOM mPyrr]­[TFSI], EO1) and, (D) (*N*-methyl-*N*-(2-(2-(2-methoxyethoxy)­ethoxy)­ethyl) pyrrolidinium bis­(trifluoromethylsulfonyl)­imide, [(EO)_3_M mPyrr]­[TFSI], EO3).

**Table 1:** Composition Abbreviations, Molar Ratio Amounts, and [O]/[Li^+^] Ratios

abbreviation	composition in molar ratios	[O]/[Li^+^] ratio
LiG4TFSI	1:1 LiTFSI:G4	5
111EO1	1:1:1 EO1:LiTFSI:G4	6
1108EO1	1:1:0.8 EO1:LiTFSI:G4	5
111EO3	1:1:1 EO3:LiTFSI:G4	8
1104EO3	1:1:0.4 EO3:LiTFSI:G4	5

## Materials and Methods

### Materials

LiTFSI (Iolitec) was stored in an argon-filled glovebox (MB200B Mbraun, H_2_O and O_2_ between <0.1 and 5 ppm). LiTFSI was dried in a vacuum oven at 120 °C for over 24 h prior to use. G4 was purchased from Sigma-Aldrich and stored in a desiccator. Prior to use, G4 was dried using molecular sieves (4 Å, Alfa Aesar) in the glovebox. The ionic liquids *N*-(2-methoxyethyl)-*N*-methyl pyrrolidinium bis­(trifluoromethylsulfonyl)­imide ([EOM mPyrr]­[TFSI]), *N*-methyl-*N*-(2-(2-(2-methoxyethoxy)­ethoxy)­ethyl) pyrrolidinium bis­(trifluoromethylsulfonyl)­imide, [(EO)_3_M mPyrr]­[TFSI] were synthesized using methods previously described by Lall-Ramnarine et al.^29^ and described further in the SI. Prior to use, the ILs were dried in a vacuum oven at 45 °C and −29 in Hg for up to 2 weeks. The dried pristine ILs were stored in the glovebox, and the water content was tested periodically to ensure the water content did not increase over time. The water content of the ILs and G4 was <50 ppm before use, measured using a coulometric Karl Fischer titrator (Metrohm 803 Ti). All electrolyte compositions were prepared in the glovebox and mixed using a magnetic stir bar for more than 24 h.

### Characterization Methods

All electrochemical experiments were performed using a Gamry Interface 1000 potentiostat. Linear sweep voltammetry (LSV) was performed using a commercial low-volume cell purchased from BASI with a sample size of 0.5 ± 0.1 mL. LSV experiments were performed in the glovebox (29 ± 2 °C). The working electrode was a 1.6 mm platinum disk polished with 0.05 μm alumina slurry before all experiments. A Pt gauze electrode (99.9%, 2.5 cm × 2.5 cm, Alfa Aesar) was used as a counter electrode, and it was flame-cleaned, followed by washing with water and drying. An Ag/Ag^+^ reference electrode was assembled using a nonaqueous reference electrode kit from BASI. The glass body was filled with 10 mM silver triflate (STREM Chemical Inc., 99%) in 1-butyl-1-methylpyrroldinium bis­(trifluoromethylsulfonyl)­imide (Sigma-Aldrich, “high purity”). Oxidation scans were performed at 20 mV/s starting at 0 V vs Ag/Ag^+^ and stopping at a current of 100 ± 20 μA (5 ± 1 mA/cm^2^). After each experiment, approximately 5 mM ferrocene was added to the electrolyte as an internal standard. Cyclic voltammetry was performed from −0.1 V to −0.9 V vs Fc/Fc^+^ at 20 mV/s for 5 scans to determine the half-wave potential. The reported potentials versus Li/Li^+^ were converted from measured Fc/Fc^+^. A cutoff current of 0.5 mA/cm^2^ was used to determine the oxidation limit.

Cyclic voltammetry was performed using a PTFE T-type union connector (McMaster-Carr, OD: 6 mm, nonbored-through) with a sample size of 0.3–0.4 mL. Experiments were conducted in an environmental chamber (BTZ133, Espec) to control temperature. The working electrode was a (3 mm) copper disk that was polished with 15, 3, and then 1 μm diamond polish slurry. The counter electrode was lithium metal pressed onto a copper rod (6 mm). A small piece of lithium metal pressed into the end of a pipette tip with nickel wire was used as a reference electrode. Potentials were scanned from −0.75 to 1.25 V vs Li/Li^+^ for 10 scans at a scan rate of 10 mV/s.

Electrochemical impedance spectroscopy (EIS) was utilized to determine the ionic conductivities of the electrolyte. A commercial conductivity cell (BioLogics HTCC) with parallel black platinized electrodes was used from 1 Hz to 100,000 Hz with perturbations of 10 mV. The cells were filled with approximately 0.6 mL of the electrolyte and sealed in the glovebox. EIS experiments were conducted in an environmental chamber (BTZ133, Espec) to control the temperature. Measurements were taken every 10 °C between −40 and 60 °C after 30 min of temperature equilibration. Conductivity was determined by using a cell constant that was experimentally determined by using a 0.01 M KCl conductivity standard (RICCA).

Thermal analysis was done using differential scanning calorimetry (DSC, TA Instruments DSC25 with an RCS90 chiller installed). Samples between 6 and 12 mg were weighed into Tzero hermetic aluminum pans and lids (TA Instruments) and crimped inside the glovebox. Samples were cooled from 30 to −85 °C at 10 °C/min and then heated to 120 °C at 10 °C/min for 3 cycles and then a final cycle with the same limits at 5 °C/min. The reported values were taken from the final cycle at 5 °C/min. The reported glass transition temperatures were acquired in TRIOS (TA Instruments) software by analyzing the change in the baseline and calculating the onset of the transition.

Viscosities were measured with a Cambridge Applied Systems ViscoLab 4000 electromagnetic reciprocating piston viscometer that was temperature regulated by a Lauda RM-6 circulating bath with a 60/40 v/v propylene glycol/water mixture. The viscometer was calibrated with S6 and S60 viscosity reference standards from Koehler Instrument Co. (Bohemia, NY). The viscometer was housed in a moisture-controlled dry box with an active N_2_ purge during data acquisition. The dry box was fitted with a hygrometer, and measurements were made when the moisture level was less than 1%. Density measurements were performed on an Anton Paar DMA 4100 M density meter. The sample chamber was cleaned with ∼5 mL washes of water, ethanol, and acetone, and then air was passed through for approximately 5 min. Air checks were passed before each sample was inserted. Measurements were taken in 10 °C increments from 70 to 10 °C.

Raman spectroscopy was performed on a WITec confocal alpha300R microscope using a 532 nm laser. Samples were prepared on a glass slide in the glovebox. The cover glass was epoxied on all sides to protect the sample from moisture before removal from the glovebox.

All liquid-state and pulsed-field gradient (PFG) NMR measurements were obtained on a Bruker AVANCE NEO 600 NMR spectrometer with a 14.1 T narrow-bore (51 mm) superconducting magnet operating at 599.76, 564.34, 233.09, and 150.81 MHz for ^1^H, ^19^F, ^7^Li, and ^13^C nuclei, respectively. A Bruker 5 mm double-resonance ^1^H–^19^F/X DiffBB probe was used for all measurements, which had a *z*-axis gradient with a maximum strength of 17 T/m. All ^1^H, ^19^F, ^7^Li, and ^13^C NMR experiments were acquired using rf field strengths of 13.8 16.6, 15.0, and 17.9 kHz, respectively, corresponding to π/2 pulses of 18.00 μs, 15.04 μs, 16.62 μs, and 14.00 μs, respectively. Samples were prepared in an argon-filled glovebox (<1 ppm of H_2_O, O_2_) in a 5 mm quartz tube, with a 3 mm coaxial tube insert containing D_2_O (99.8%) for solvent locking. The NMR tubes were sealed in a glovebox using epoxy to prevent sample exposure to air during measurements. Liquid-state ^1^H, ^19^F, ^7^Li, and ^13^C single-pulse NMR spectra were acquired at 25 °C under quantitative conditions using recycle delays such that the nuclear spin systems relaxed to thermal equilibrium between scans. All liquid-state ^13^C single-pulse NMR experiments were acquired under heteronuclear ^1^H–^13^C decoupling by using the WALTZ-65 pulse scheme. All ^1^H, ^7^Li, and ^19^F PFG-NMR measurements of self-diffusion coefficients were conducted using a stimulated-echo pulse sequence using bipolar pulse gradient pairs, sinusoidal gradient pulses, spoiler gradients, and longitudinal eddy decay delays (5 ms). Experiments were conducted using a range of diffusion times (Δ, 250–500 ms), gradient pulse lengths (δ, 2.0 ms), and gradient strengths (300–800 G/cm). All PFG-NMR diffusion experiments were performed at 30 °C, enabling direct comparisons to the conductivity measurements.

All density functional theory (DFT) calculations were performed using the Gaussian 16 software package.^48^ The B3LYP hybrid exchange–correlation functional, in combination with the 6–311G+(d) basis set, was employed throughout. Initial molecular structures were constructed using Avogadro2 and preoptimized using the MMFF94 force field.^49^ These geometries were subsequently used as starting points for full geometry optimizations in Gaussian. Following geometry optimization, frequency calculations were conducted to confirm the nature of the stationary points and to obtain the thermodynamic data. Only structures with no imaginary frequencies were considered for further analysis. The interaction energy between the Li^+^ cation and a ligand (G4, EO1, and EO3) was evaluated in both the gas phase and using a polarizable continuum model (PCM) to account for solvation effects. To approximate the dielectric environment of glymes and ionic liquids, a solvent with an approximate dielectric constant (ε) of 10 was used, selected based on literature values.^50,51^

## Results and Discussion

### Physical and Mass Transport Properties of the Ternary Electrolytes

We sought to investigate how the addition of the ether-functionalized ILs to LiG4TFSI would affect the thermal, electrochemical, and physicochemical properties of the resulting electrolyte mixtures. As a preliminary screening, the phase behavior and oxidative stability were first measured. The purpose of these preliminary experiments was to reject electrolyte candidates that would not be applicable below ambient temperatures or when paired with high-voltage cathode materials. The ternary mixtures did not limit the low temperature liquid range compared to LiG4TFSI (Figure S1). The glass transition temperature of LiG4TFSI was −58.9 °C. The glass transitions of 111EO1 and 1108EO1 compositions both shifted to lower temperatures of −75.1 and −73.0 °C, respectively. In the 111EO3 composition, the glass transition also shifted to a lower temperature of −66.3 °C, while the 1104EO3 composition shifted slightly higher to −51.0 °C.

The oxidative stability limit was measured to determine the impact of the IL additives on ether oxidation (Figure S2). A current density threshold of 0.5 mA/cm^2^ was used to determine the oxidation limits. The LiG4TFSI and 111EO1 compositions have similar oxidative limits of 5.25 and 5.23 V, respectively, while the 1108EO1 composition exhibited a higher limit by 50 mV compared to LiG4TFSI. In EO3 compositions, the 111EO3 had a lower oxidative limit compared to LiG4TFSI by 33 mV, and the 1104EO3 composition was 80 mV higher. Suppression of the ether oxidation onset potential was found at 4.4 V versus Li/Li^+^ in compositions with [O]/[Li^+^] = 5. It is important to note these are not optimized compositions for the bulk physiochemical and electrochemical properties. The compositions were chosen to study the effect of the EO1 and EO3 cations as well as the [O]/[Li^+^] ratio on the properties and cation complex of LiG4TFSI.

The conductivities and viscosities of the electrolyte were evaluated across wide temperature ranges. Figure 2A shows the temperature-dependent conductivity measurements from −40 to 60 °C. The ternary mixtures had higher or similar conductivity through the entire temperature range compared to LiG4TFSI, except for the 1104EO3 composition. At 30 °C, the conductivity of LiG4TFSI was 1.53 mS/cm. Both the 111EO1 and 1108EO1 compositions had higher conductivity of 2.54 mS/cm and 1.85 mS/cm at 30 °C compared to LiG4TFSI. The 111EO3 and 1104EO3 compositions had a conductivity of 1.37 mS/cm and 0.279 mS/cm at 30 °C. Figure 2B shows the viscosity measured from 0 to 95 °C (tabulated values in Table S2). As expected, the viscosity data followed the opposite trend to the conductivity. The measured viscosity of LiG4TFSI was 88.7 cP at 30 °C. The 111EO1 and 1108EO1 compositions had viscosities of 59.3 cP and 93.8 cP, respectively, at 30 °C. The 111EO3 and 1104EO3 compositions had viscosities of 83.1 cP and 506.4 cP, respectively, at 30 °C. For both the conductivity and viscosity, the [O]/[Li^+^] ratio had a significant impact, indicating the importance of the G4 coordination complex with Li^+^. When [O]/[Li^+^] = 5, the conductivity tends to be lower, and the viscosity is higher compared to [O]/[Li^+^] > 5 compositions with the same IL. As temperature decreases, we also observed the EO1 ternary mixtures maintain a higher conductivity and lower viscosity compared to LiG4TFSI. The measured viscosity of LiG4TFSI at 25 °C was 110 cP, and from the literature, the values for the EO1 and EO3 TFSI ILs at 25 °C^29^ are 54 cP and 64 cP, respectively. The viscosity of the 1104EO3 composition is higher by approximately an order of magnitude compared to LiG4TFSI and the other ternary composition and was significantly higher than the individual components. When considering the viscosities of the individual components and ternary mixtures, we hypothesize that the more significant change in the liquid structure between the 111EO3 and 1104EO3 compositions than between the 111EO1 and 1108EO1 compositions is due to increased ionic and intermolecular interactions between the Li^+^ and the EO3 side chain in the G4-deficient 1104EO3 composition where [O]/[Li^+^] = 5. The higher concentration of the TFSI^–^ anion in the G4-deficient 1104EO3 composition will also contribute to the significant increase in viscosity due to an increase in ion cluster and aggregation facilitated by TFSI^–^.^52^

**Figure 2:**
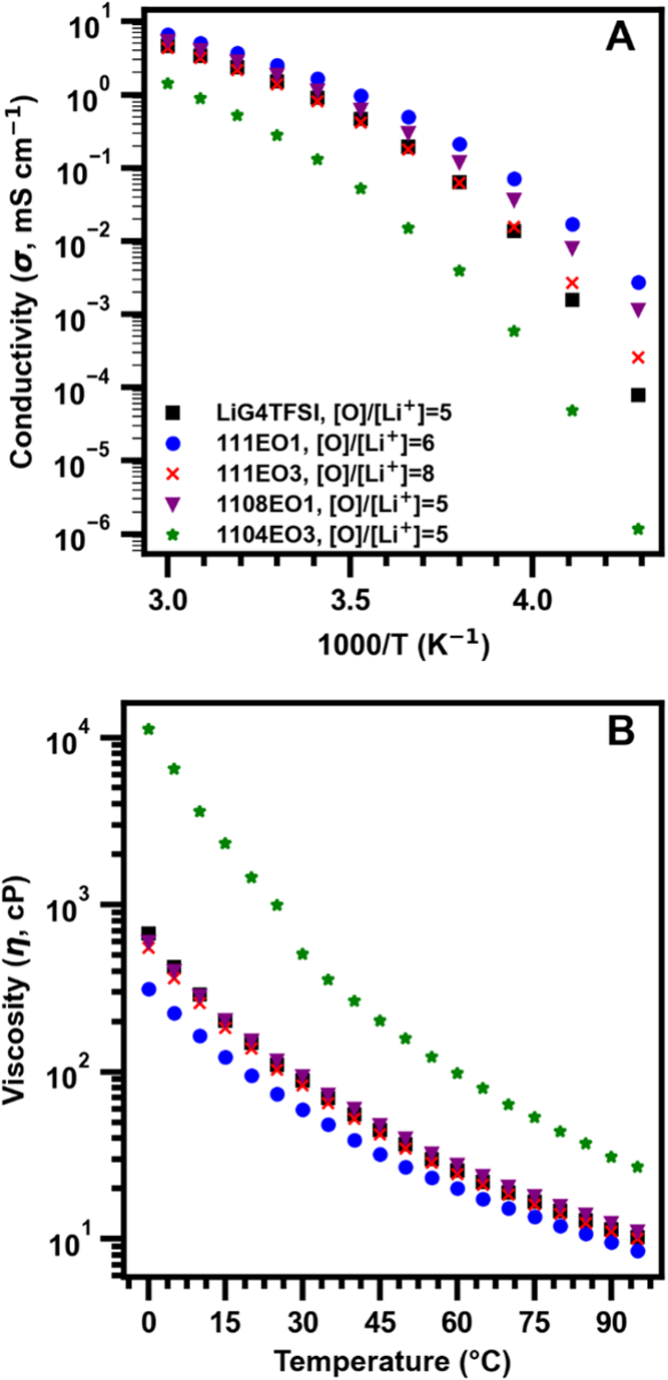
(A) Conductivity of electrolyte compositions measured from −40 to 60 °C. (B) Viscosity of electrolyte compositions measured from 0 to 95 °C.

A Walden plot was constructed (Figure 3) to better understand how the measured conductivity and fluidity of the electrolyte deviate from the Nernst–Einstein relationship. An IL that is close to the KCl line is “ideal” and considered a “good” IL where ions are dissociated. As ILs deviate further below the KCl line, they are considered “subionic” or “poor” ILs and can be associated with lower conductivity.^53^ We acknowledge that the 1.0 M KCl line as an arbitrary reference point for IL-based systems and Walden Plots have limited physical meaning in relation to the transport properties and ionicity of the electrolyte.^54,55^ eqs S1–S3 were used to calculate the molar conductivity (Λ_imp_) at 30 °C from the measured conductivity and density. With the Walden plot limitations in mind, the ternary electrolyte mixtures show similar behavior to LiG4TFSI, suggesting that the addition of the IL does not severely impact the conductivity–fluidity relationship. The inset shows subtle changes dictated by the IL cation rather than the [O]/[Li^+^] ratio. The Walden product, the product of the molar conductivity and viscosity, may be used as a quantitative measure of ionicity, assuming it remains constant for a particular fluid.^56^ The Walden product for LiG4TFSI is 0.495 P S cm^2^ mol^–1^ at 30 °C. The Walden product values for the 111EO1 and 1108EO1 compositions are 0.496 P S cm^2^ mol^–1^ and 0.537 P S cm^2^ mol^–1^, respectively. Walden products for the 111EO3 and 1104EO3 compositions had the lowest values of 0.418 P S cm^2^ mol^–1^ and 0.424 P S cm^2^ mol^–1^, respectively. The Walden products of the pure IL decrease with an increase in chain length.^29^ The trend with the pure EO1 and EO3 ILs is consistent with the EO1 and EO3 ternary compositions.

**Figure 3:**
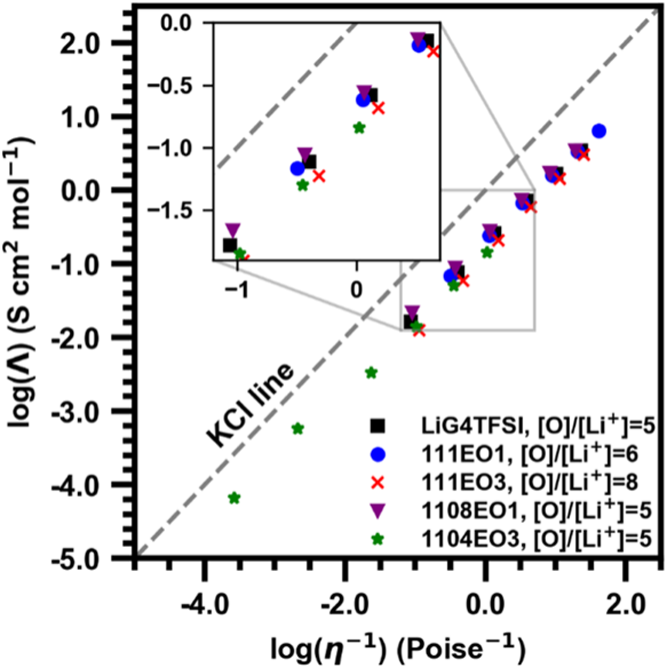
Walden plot of compositions from 10 to 70 °C, where the dashed gray line is the ‘KCl’ ideality line.

To understand ion mass transport properties in the ternary electrolytes, multinuclear PFG-NMR measurements were used to quantify and compare the diffusion coefficients of each species. The diffusion coefficients for tetraglyme, the ether-functionalized pyrrolidinium cation (EO1 or EO3), the TFSI^–^ anion, and the Li^+^ cation for each composition were measured using their ^1^H, ^19^F, and ^7^Li signals, respectively (Figure 4A). The [O]/[Li^+^] > 5 ternary compositions, 111EO1 and 111EO3, exhibited faster diffusion of all constituent species compared to the [O]/[Li^+^] = 5 compositions, 1108EO1 and 1104EO3. The EO1 IL cation had the highest diffusion coefficient in the 111EO1 and 1108EO1 compositions, suggesting that it does not strongly interact with other species.

**Figure 4:**
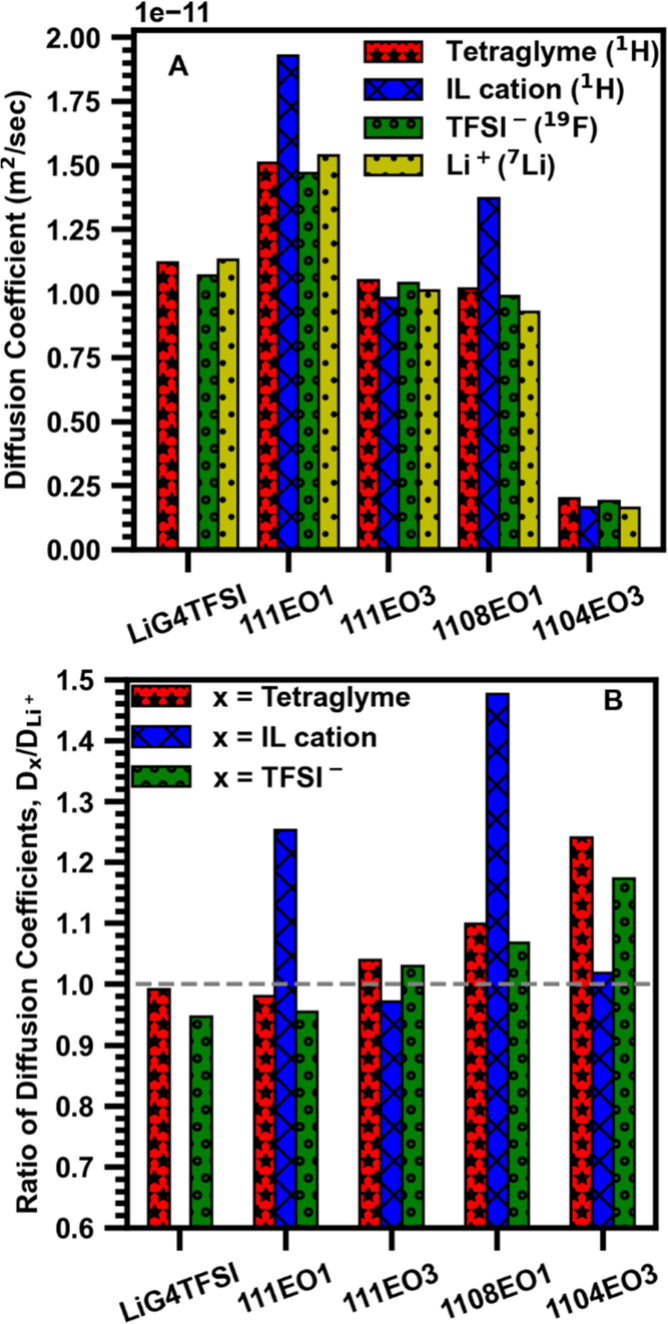
Multinuclear PFG-NMR measurements of the (A) diffusion coefficients of each nucleus and (B) ratio of diffusion coefficients D_*x*_/D_Li+_, where *x* is tetraglyme (G4), the IL cation, or the TFSI^–^ anion.

The ratios of diffusion coefficients of each species were compared with the diffusion coefficients of Li^+^ cations within that composition. The ratios D_*x*_/D_Li_^+^ (Figure 4B) yield insights into the coordination complex of the Li^+^ cation in these solvate ionic liquid systems. LiG4TFSI had a D_G4_/D_Li_^+^ approximately equal to 1, which indicates that the Li^+^ cation and tetraglyme molecule diffuse as a coordinated complex.^57^ In the EO1 compositions, EO1 has a significantly higher diffusion coefficient than Li^+^. In contrast, the EO3 compositions exhibit a D_EO3_/D_Li+_ ratio closer to 1, which suggests that the EO3 IL cation has stronger interactions with Li^+^ cations. This finding serves as evidence that EO3 better coordinates to the Li^+^ cation than the shorter chain EO1, a result that is corroborated below by liquid-state ^13^C single-pulse NMR measurements.

To better understand ion interactions, we analyzed the ionicities of the electrolyte mixtures by comparing the experimental and theoretical molar conductivities, assuming complete ion dissociation. To do so, we computed the inverse Haven ratio, Λ_imp_/Λ_NMR_, where Λ_imp_ is the molar conductivity determined from electrochemical impedance spectroscopy and density measurements and Λ_NMR_ (eq 1) is the molar conductivity calculated using the Nernst–Einstein relationship^58,59^

1 $$\Lambda_{\mathrm{NMR}}=\frac{N_{a}e^{2}}{\kappa T}\left(\left[\chi_{Li^{+}}D_{Li^{+}}+\chi_{\mathrm{catio}n^{+}}D_{\mathrm{catio}n^{+}}\right]+D_{\mathrm{TFS}I^{-}}\right)$$

where *N*_a_ is Avogadro’s number, *e* is the elementary charge constant, κ is the Boltzmann constant, *T* is the temperature in Kelvin, χ is a mole fraction, and *D*_*x*_ is the diffusion coefficient of each species *x* determined from PFG-NMR measurements. The inverse Haven ratio Λ_imp_/Λ_NMR_ represents the fraction of charged species contributing to ion conduction.^60^ A Λ_imp_/Λ_NMR_ ratio of 1 indicates that an electrolyte is ‘ideal’ and all ions are completely dissociated because the implicitly measured conductivity agrees with the Nernst–Einstein relationship assumptions made to determine Λ_NMR_. This ratio, as well as the Walden Plot (Figure 3), are both concepts related to “ionicity” or ion dissociation.^58,61^ The Λ_imp_/Λ_NMR_ ratio for LiG4TFSI was 0.69. For the 111EO1 and 1108EO1 compositions, the values were 0.71 and 0.72, respectively. The values for the 111EO3 and 1104EO3 compositions were 0.67 and 0.64, respectively. The Λ_imp_/Λ_NMR_ ratio shows a trend consistent with the Walden products, where the values for all EO1 compositions are greater than EO3 compositions and LiG4TFSI. We observe a slight difference in trends when comparing the 111EO3 and 1104EO3 compositions. The inverse Haven ratio gives a higher value for 111EO3, and the Walden product gives a higher value for 1104EO3. An explanation could be the effect of the significantly higher viscosity in the 1104EO3 that is accounted for in the Walden product. Overall, these findings indicate greater ion dissociation in the EO1 mixtures compared to the EO3 compositions. (Figure S4).

The lithium transfer number (*t*_Li^+^_) represents the fraction of the total current carried by Li^+^ through the electrolyte in the absence of concentration gradients. Approximating *t*_Li^+^_ is often done by measuring the diffusion coefficients from PFG-NMR measurements. The transference number *t*_Li^+^_ is determined using eq 2

2 $$t_{Li^{+}}=\frac{\chi_{Li^{+}}D_{Li^{+}}}{\chi_{Li^{+}}D_{Li^{+}}+\chi_{IL^{+}}D_{IL^{+}}+\chi_{\mathrm{TFS}I^{-}}D_{\mathrm{TFS}I^{-}}}$$

χ is a mole fraction, and *D*_*x*_ is the diffusion coefficient of each species *x* determined from PFG-NMR measurements. This method is limited because the approximation of *t*_Li^+^_ does not account for anticorrelated motion.^62^ However, its calculation is useful as an additional basis of comparison between the different electrolyte compositions. The calculated *t*_Li^+^_ value of LiG4TFSI was determined to be 0.51, which agrees with previous literature,^63,64^ For 111EO1 and 1108EO1, the transference numbers were 0.24 and 0.22, respectively, while the 111EO3 and 1104EO3 values were 0.25 and 0.23. When the EO1 and EO3 ILs are taken into account, *t*_Li^+^_ decreases significantly. The ternary mixtures themselves yield similar *t*_Li^+^_ values, though for the [O]/[Li^+^] = 5 mixtures, 1108EO1 and 1104EO3 have lower *t*_Li^+^_ numbers than 111EO1 and 111EO3, respectively. The EO3 ternary mixtures also show slightly higher *t*_Li^+^_ numbers than the EO1 mixtures due to the high D_EO1_ values.

To demonstrate and compare reversible electroplating and electrostripping of lithium between the electrolyte compositions, cyclic voltammetry was performed and used to calculate Coulombic efficiencies (Figure S3). A 3-electrode liquid cell using a PTFE T-type connector union was used with a Cu working electrode and lithium counter, as described in the Materials and Methods Section, to evaluate reversible plating and stripping behavior as well as determine the influence of interfacial layer formation. All electrolyte compositions could reversibly electroplate and electrostrip lithium metal. LiG4TFSI exhibited a Coulombic efficiency of 60.2% in cycle 1, which increased to 82.4% at cycle 10. The 1104EO3 composition had a Coulombic efficiency of 49.5% at cycle 1, which improved to 58.3% at cycle 10. The 1108EO1 composition had a Coulombic efficiency of 46.3% at cycle 1 and hit a maximum at cycle 59.7% at cycle 5 before decreasing to 56.1% at cycle 10. Both the 111EO1 and 111O3 compositions showed significantly lower Coulombic efficiencies than LiG4TFSI. The 111EO3 composition started at cycle 1 with 53.3% and steadily decreased to 25.5% at cycle 10. The 111EO1 composition remained below 25% through all cycles. The [O]/[Li^+^] ratio had a significant effect on the interfacial stability in the ternary electrolytes. The lower columbic efficiencies for the [O]/[Li^+^] > 5 electrolytes could be due to the excess glyme decomposition affecting the formation of stable interfaces.

To test how the formation of a stable interface impacted the plating and stripping, Coulombic efficiencies were determined for both LiG4TFSI and the 1108EO1 composition with and without 4% (w/w) fluorinated ethylene carbonate (FEC) (Figure S4). FEC is commonly used as an additive to form a robust SEI and improve cycling stability.^24,65,66^ Shobukawa et al. found that adding FEC to LiG4TFSI improved cycling stability and transport properties without impacting the solvation structure.^67^ In our cyclic voltammetry experiments, the Coulombic efficiencies significantly improved with the addition of FEC. For LiG4TFSI + 4% FEC, cycle 1 had a Coulombic efficiency of 74.0% and improved to 79.0% at cycle 10. The 1108EO1 composition had a Coulombic efficiency of 81.8% at cycle 1 and decreased to 76.3% at cycle 10. The FEC additive results show the significance of the initial interfacial formation during lithium deposition on copper and further support that the solvation structure of compositions without FEC has a profound impact on interfacial stability.

### Spectroscopic and DFT Analyses of Coordination Structures

For LiG4TFSI, the stability of the Li­[G4]^+^ cation complex has been studied due to its relation to electrolyte properties, including electrochemical stability, Li^+^ transport, and ionicity.^6,68^ Here, we utilized both Raman and NMR spectroscopy to elucidate changes in the Li^+^ solvation complex with the addition of solvating ILs.

Raman spectroscopy can be used to probe the local solvation complex of SILs.^69,70^ The bands at ∼740 cm^–1^ are assigned to coupled CF_3_ bending modes and S–N stretching of the TFSI^–^.^71^ These bands are very sensitive to Li-TFSI interactions, and distinguishing between solvent-separated ion pairing (SSIP) and contact ion pairing (CIP) of the TFSI^–^ anions has been reported in the literature.^72^ The LiTFSI salt has a peak at 747 cm^–1^, representing the CIP of the Li^+^ and TFSI^–^.The Li­[G4]^+^ complex causes a shift to a lower wavenumber due to the tetraglyme occupying the primary solvation shell of Li^+^ and an SSIP interaction with the TFSI^–^.^73^ Figure 5 shows our experimental data of the TFSI^–^ S–N stretching region. This region was fitted with a Pseudo-Voigt function to deconvolute peaks at ∼746 cm^–1^ and ∼741 cm^–1^, representing CIP and SSIP, respectively. Only assigning two peaks to the TFSI^–^ S–N stretch in Raman is common but is under scrutiny.^74^ We acknowledge that CIP conformations of TFSI^–^ may overlap with the assigned SSIP peak and are not accounted for in this analysis. The integral areas were thus used to calculate the fraction of CIP to SSIP in each composition. The area_CIP_/area_SSIP_ fraction was 0.255 for LiG4TFSI. For the pure EO1 and EO3 ILs, the fractions were 0.031 and 0.034, respectively. The low CIP contributions in the pure ILs mean that the ternary mixture CIP should come from interactions between the Li^+^ and the TFSI^–^ anion. For compositions 111EO1 and 111EO3 where [O]/[Li^+^] > 5, the fractions are 0.105 and 0.132, respectively. The integrated areas suggest a greater fraction of SSIP in 111EO1 and 111EO3 compositions compared to LiG4TFSI. The values for the [O]/[Li^+^] = 5 compositions, 1108EO1 and 1104EO3, are 0.323 and 0.271, respectively. Both compositions had a lower molar amount of G4 than that of Li^+^. The 1108EO1 composition has 20% less G4 than 111EO1, and the 1104EO3 has 60% less G4 than 111EO3. The higher fraction of CIP in the 1108EO1 composition may be due to the lower molar amount of G4 and the EO1 cation not interacting with the Li^+^. In contrast, the 1104EO3 composition has a comparable fraction of SSIP and CIP to LiG4TFSI and significantly less G4 than the 1108EO1 composition, which suggests EO3 side chain interactions with Li^+^, maintaining the SSIP between Li^+^ and TFSI^–^. The area_CIP_/area_SSIP_ values, as well as fitted peak positions, are provided in Figure S5.

**Figure 5:**
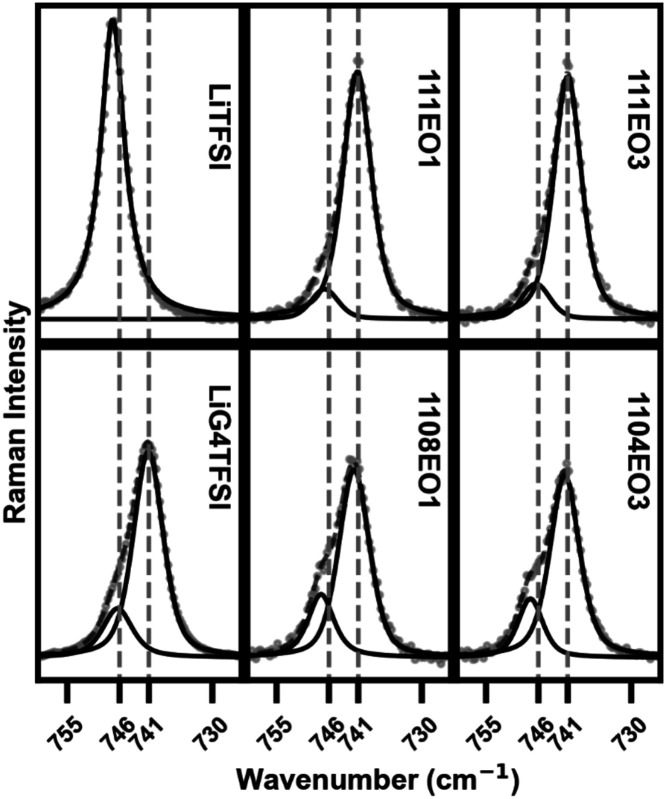
Raman spectra of the CF_3_ bending and S–N stretch of the TFSI for each composition.

To better understand interactions of the G4 and EO3 cation, the range between 770 and 900 cm^–1^, assigned to CH_2_ bending and C–O–C bending modes, is analyzed for select compositions. Upon coordination to Li^+^, a strong peak at 868 cm^–1^ appears, which represents a breathing mode from the formation of the Li­[G4]^+^ cation complex. In Figure 6, we observe the expected formation of the G4 breathing mode between G4 and LiG4TFSI. To better understand the behavior of the EO3 ether-functionalized side chain, we compared the neat IL and 1:0.6 EO3:LiTFSI ([O]/[Li^+^] = 5) compositions without G4 added. Upon addition of LiTFSI to neat EO3, we observe the formation of a peak ∼870 cm^–1^, which we attribute to the interaction of the side chain with Li^+^. This peak also features a shoulder of ∼877 cm^–1^ that is observable in the 1104EO3 composition. These findings suggest that the EO3 side chain interacts with Li^+^ in the G4-deficient 1104EO3 composition.

**Figure 6:**
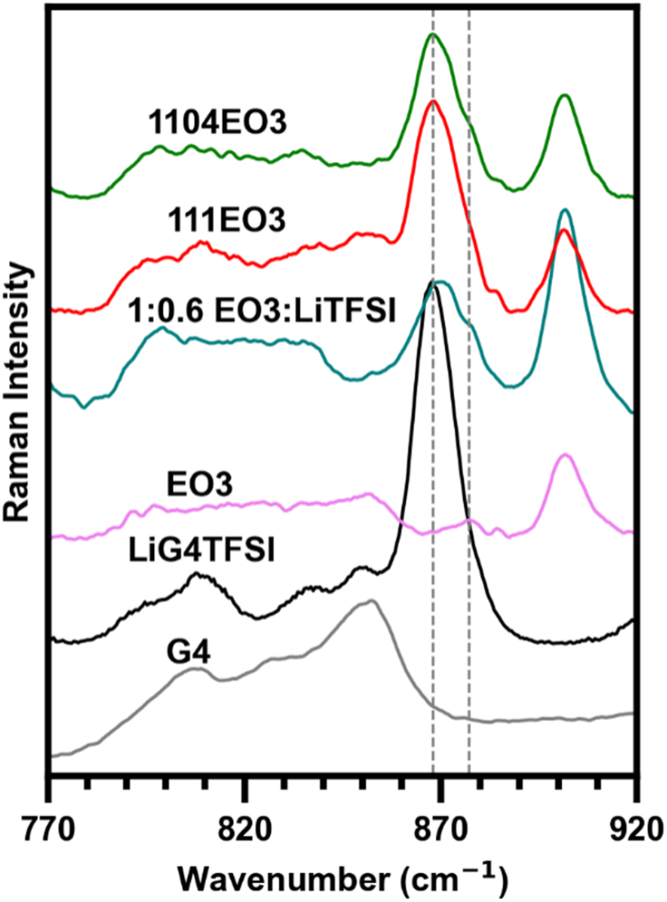
Raman spectra of the C–O–C bending and breathing mode of select compositions; dashed lines are guide for the eyes at 868 cm^–1^ and 877 cm^–1^.

The electrolyte compositions were further examined by analyzing their liquid-state ^13^C NMR single-pulse NMR spectra. Changes in NMR chemical shifts enable changes in the local electronic environments to be probed for the different nuclei, including how they change between the various compositions. Due to the larger chemical shift range of ^13^C nuclei, the ^13^C NMR spectra were analyzed here, where changes in ^13^C chemical shift were used to analyze changes in intermolecular interactions and coordination with molecular-level specificity. The ^1^H, ^7^Li, and ^19^F, single-pulse NMR spectra are shown in Figures S5–S7, respectively.

The liquid-state ^13^C single-pulse NMR spectra of the EO1 and EO3 compositions, as well as their constituent components, are shown in Figures 7A and 8A, respectively. The differences in the ^13^C chemical shifts for each ^13^C position in tetraglyme, with respect to pure tetraglyme, are plotted in Figures 7B and 8B. Similarly, the differences in ^13^C chemical shifts for each ^13^C position in the IL cation, with respect to that of the pure IL cation, are plotted in Figures 7C and 8C. Molecular structures with ^13^C signal assignments are shown in Figures 7B,C and 8B,C. In addition to the ternary mixtures, G4EO1 and G4EO3 binary mixtures containing no Li^+^ were utilized to better understand the effect of the solvent interactions.

**Figure 7:**
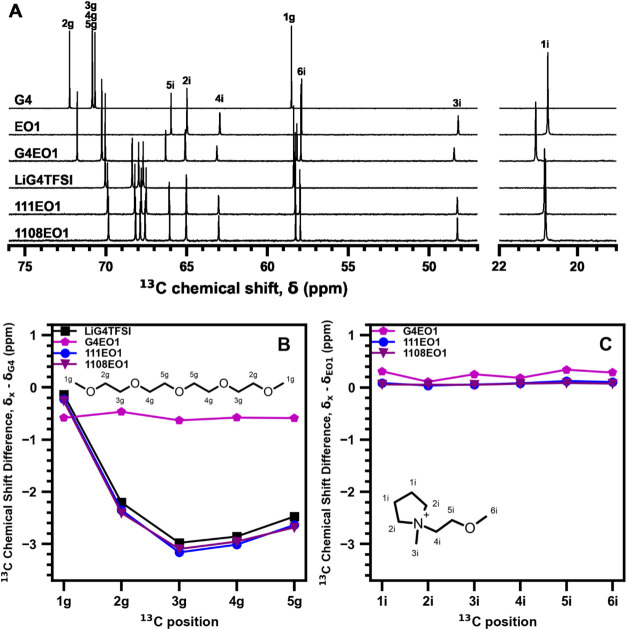
(A) Liquid-state ^13^C single-pulse NMR spectra for the EO1-containing composition and constituent components with ^13^C signal assignments. (B) Difference in ^13^C chemical shifts of the tetraglyme in each mixture, for each carbon position, is compared to pure tetraglyme. (C) Difference in ^13^C chemical shifts of the EO1 cation in each composition, for each carbon position, is compared to that of pure EO1.

**Figure 8:**
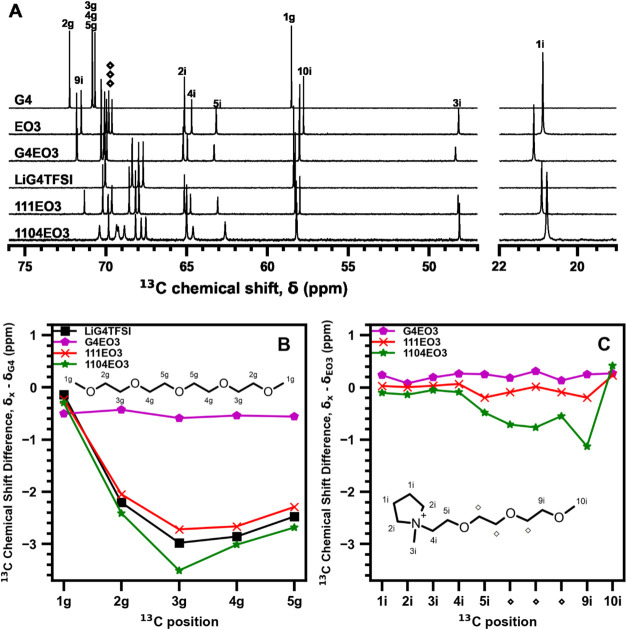
(A) Liquid-state ^13^C single-pulse NMR spectra for the EO3-containing composition and constituent components with ^13^C signal assignments. (B) Difference in ^13^C chemical shifts of the tetraglyme in each mixture, for each carbon position, compared to pure tetraglyme. (C) Difference in ^13^C chemical shifts of the EO3 cation in each composition, for each carbon position, is compared to that of pure EO3.

As shown in Figure 7B, the ^13^C signals associated with CH_2_ moieties in tetraglyme shift to a lower frequency upon the addition of Li^+^ cations. The terminal CH_3_ carbons exhibited only subtle ^13^C shifts, as expected. The changes in ^13^C chemical shift upon Li^+^ cation addition is evidence of the coordination of the G4 ether oxygens with Li^+^ cations. This shift to lower frequency establishes that greater electron density (and hence shielding) is observed on the tetraglyme CH_2_ carbon moieties upon coordination to Li^+^ cations, which may be counterintuitive to what would be expected from a coordinating ligand that can donate electron density to the cation. An explanation to this observation has been reported in the literature by Black et al.,^75^ who used NMR spectroscopy to show that the TFSI^–^ anion counteracts electron donation from the G4 oxygen atoms to the Li^+^ cations. Thus, the TFSI^–^ anions appear to play an indirect but measurable role in the electronic environments of the Li­[G4]^+^ cation complex. Without the addition of Li^+^ cations, there is a much weaker and approximately equal ^13^C shift to a lower frequency for all carbon positions G4. The minimal ^13^C shift in the G4EO1 composition without Li^+^ cations further demonstrates that the large shifts observed in the 111EO1 and 1108EO1 compositions are due to the coordination of Li^+^ cations with the ether oxygen atoms of the tetraglyme. Interestingly, Figure 7C shows that the EO1 cation does not exhibit significant ^13^C shifts when Li^+^ ions are present. The short ether side chain of the EO1 cation therefore does not interact with the Li^+^ cations, perhaps due to electrostatic repulsions between the pyrrolidinium and Li^+^ cations.

In Figure 8B, the EO3 compositions exhibited a similar trend when comparing the tetraglyme in the EO1 and EO3 mixtures, wherein the ^13^C chemical shifts of CH_2_ moieties shifted to lower frequencies in the presence of Li^+^ cations. Similarly, the ^13^C signals for the CH_3_ groups were relatively unchanged due to Li^+^ addition, while the ^13^C signals of tetraglyme only shifted slightly to lower frequencies. Interestingly, there was a measurable difference between the ^13^C chemical shifts of the CH_2_ moieties between the 111EO3 and 1104EO3 compositions, suggesting that the EO3 cation influenced the solvation environment when less tetraglyme was added. Figure 8C also shows differences in ^13^C chemical shifts of the IL cation among the EO3 compositions, in contrast with the EO1 compositions. The 1104EO3 composition exhibited the largest lower frequency shifts, particularly on the carbon atoms of the ether side chain, though this shift is less than what we observe in G4. The ^13^C shifts observed for tetraglyme in the EO1 and EO3 compositions suggest that the Li­[G4]^+^ cation complex is not affected by the addition of the ILs. The compositions without LiTFSI show that tetraglyme does not interact as strongly with the IL cation as it does with the Li^+^ cation. The lower ^13^C frequency shifts observed for the EO1 cation suggest that the EO1 cation does not interact with the Li­[G4]^+^ complex and instead acts as a diluent. In contrast, the ether oxygen atoms in the EO3 cation of the 1104EO3 composition exhibit ^13^C shifts of approximately 1 ppm, which suggests that there are interactions between the side chain of EO3 and Li^+^ cations in the G4-deficient composition. The interaction of Li^+^ cations and ether-functionalized ILs has previously been investigated. ^1^H–^7^Li HOESY NMR analysis from Zamory et al.^20^ showed that the EO1 cation has the closest proximity to the Li^+^ cation at hydrogen neighboring the nitrogen of the pyrrolidinium ring due to the localized structure facilitated by strong interactions between TFSI^–^ anions and the cations. The EO3 cation ether side chain coordinates with the Li^+^ cation, which has the strongest spatial proximity to the center oxygen of the side chain.

To gain further insights into the different local ion-ligand environments revealed from NMR spectroscopy, we performed DFT calculations to evaluate the interaction energies between the Li^+^ cation and coordinating ligands G4, EO1, and EO3. These calculations offer a molecular-level understanding of relative binding strengths and coordination preferences, providing insight into the trends observed experimentally. The optimized structures of the Li^+^ complexes are shown in Figure 9, and the corresponding interaction energies are summarized in Table 2. For the Li–G4 complex, the initial geometry was based on the fully coordinated structure in which all five oxygen atoms are bound to the Li^+^ ion, as this configuration has been previously established as the most thermodynamically stable.^73,76^ The coordination energies for this complex were the lowest among all systems studied when calculated in the gas phase or when using a solvent (ε = 10) in the polarizable continuum model, indicating that Li^+^ exhibits the strongest interaction with tetraglyme. In contrast, the Li–EO1 complex showed positive coordination energies, suggesting that binding between Li^+^ and EO1 is thermodynamically unfavorable under the conditions modeled. In this complex, the Li^+^ cation is in the molecular proximity of the quaternary nitrogen atom of EO1, whose electrostatic repulsions may contribute to its overall instability. Notably, this observation is consistent with the NMR results, which indicated negligible interaction between EO1 and the Li^+^ cation. As expected, the coordination energies differed markedly between the gas-phase and PCM-modeled environments since gas-phase calculations often overestimate binding strengths due to the absence of solvation effects.^73,77^

**Table 2:** Calculated Interaction Energies between a Li^+^ Cation and G4, EO1, and EO3

complex	Δ*E*_coord_ kJ mol^–1^ vacuum	Δ*E*_coord_ kJ mol^–1^ solvent (ε = 10)
Li-G4	–441.35	–89.95
Li-EO1	139.90	12.82
Li-EO3(1)	25.36	4.95
Li-EO3(2)	–84.01	–30.07
Li-EO3(3)	–93.70	–32.12

**Figure 9:**
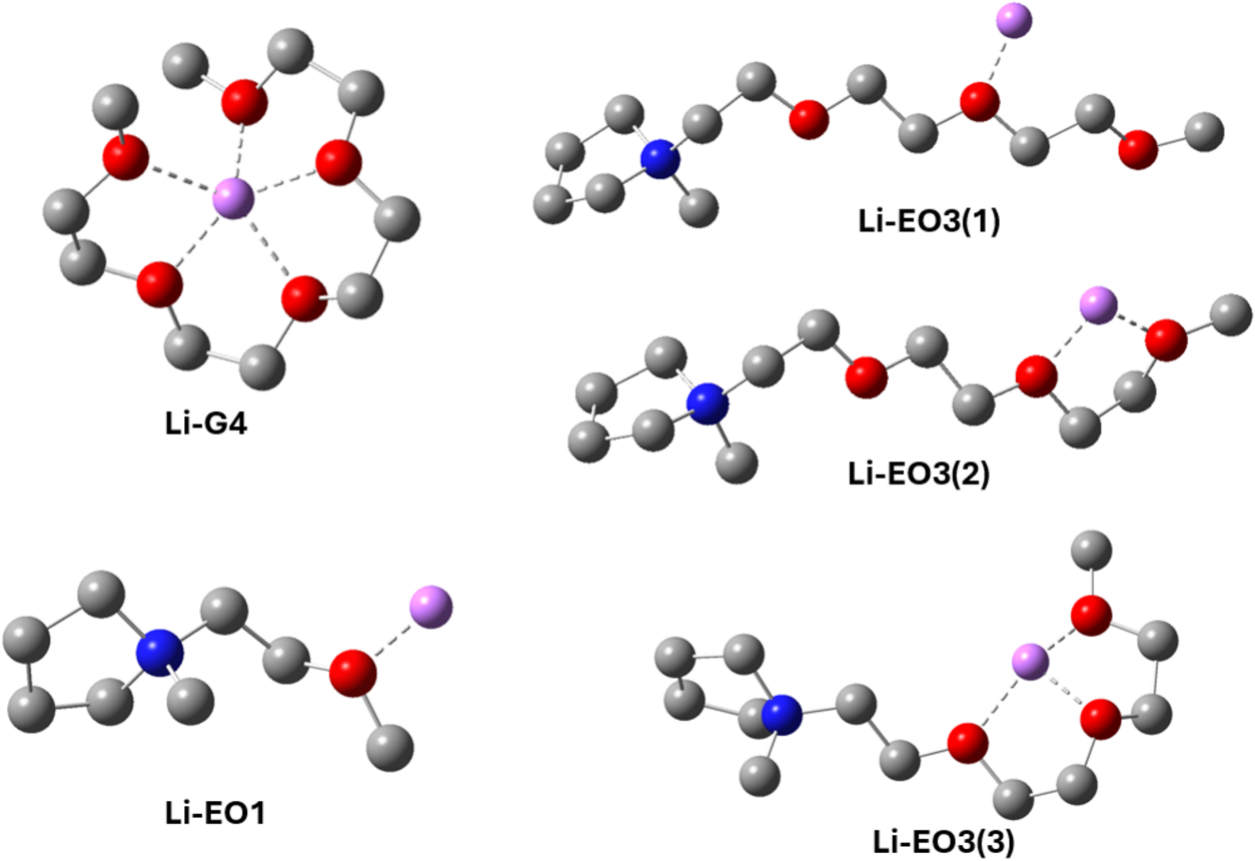
DFT-optimized structures of Li^+^ cation complexes with G4, EO1, and distinct binding configurations of EO3.

For the Li^+^-EO3 complex, three coordination configurations—monodentate, bidentate, and tridentate—were examined to capture the potential binding modes of the EO3 ligand with the Li^+^ cation. The monodentate configuration exhibited positive coordination energies in both the gas-phase and PCM environments, indicating that it is not a favorable binding mode. In contrast, both the bidentate and tridentate configurations showed negative coordination energies, suggesting that EO3 can effectively coordinate with Li^+^ when multiple donor atoms are involved. The calculated energies for the bidentate and tridentate configurations were relatively close, with the tridentate mode being only slightly more favorable. Although the Li–G4 complex exhibited the lowest coordination energy overall, confirming that tetraglyme binds most strongly to Li^+^, the ability of EO3 to bind in multidentate modes suggests that it may contribute to Li^+^ coordination in glyme-deficient compositions, as supported by NMR observations.

By investigation of the electrolyte properties and probing of the Li^+^ solvation environment, this work illustrates how functionalized ILs behave in ternary electrolyte mixtures containing G4. This work focused on molecular-level interactions, but it is important to acknowledge the importance of ion clustering and aggregation in highly concentrated and IL electrolyte systems. Further work could inform on how tuning the IL functionality of these complex ternary mixtures may impact the liquid structure of the electrolyte. The Li^+^ solvation structures elucidated by the liquid-state ^13^C single-pulse NMR experiments yield insights into the differences in the physical and transport properties measured above. Li^+^ cations preferentially coordinate with tetraglyme over ether-functionalized pyrrolidinium cations. The addition of the ether-functionalized pyrrolidinium cations does not significantly disrupt the Li­[G4]^+^ complex and furthermore only appears to coordinate with Li^+^ cations when (i) the ether side chain is sufficiently long (i.e., 3 vs 1 ether moieties) and (ii) there is insufficient glyme available to complex with the Li^+^ cations. The TFSI^–^ is a low Lewis basicity anion and weakly coordinates Li^+^, which enables the formation of the Li­[G4]^+^ complex. Choosing anions with greater Lewis basicity will impact Li­[G4]^+^ interactions and Li^+^ transport parameters, as strong Li^+^-anion interactions can disrupt the Li­[G4]^+^ cation complex.^78^ We hypothesize that the ability of this anion to interact with Li^+^ through SSIP with coordinated G4 plays an important role in stabilizing the Li­[G4]^+^ complex in these ternary mixtures.

## Conclusions

The physical and transport properties of lithium-glyme electrolytes were investigated upon the addition of ether-functionalized pyrrolidinium-based ionic liquids. We found that the EO1 cation generally increases the conductivity of the electrolyte as well as lowers the viscosity. Through PFG-NMR measurements, we observe that the EO1 cation is the fastest-diffusing species in the 111EO1 and 1108EO1 compositions, suggesting that the short ether side chain does not interact with the Li­[G4]^+^ complex. This finding is supported by liquid-state ^13^C single-pulse NMR measurements, where negligible ^13^C shifts are observed in the EO1 compositions compared to the neat EO1, as well as Raman spectroscopy, where a significant increase in contact ion pairing with the TFSI-anion occurs with partial removal of G4. In contrast, the EO3 cation has a similar diffusion coefficient to Li^+^ cations, and we measure significant ^13^C chemical shift differences in the ether side chain of the cation compared to pure G4. These observations suggest that the longer EO3 does interact with Li^+^ cations through the ether side chain, but there is no indication that it disrupts the Li­[G4]^+^ complex. DFT calculations of the interaction energies support the experimental observations. The Li-G4 complex is energetically favorable, and the Li-EO3 multidentate complexes are low complexes supporting the coordination of EO3 to Li^+^ in glyme-deficient systems. We hypothesize that the TFSI^–^ anion interaction with coordinated G4 plays an important role in stabilizing the Li­[G4]^+^ complex. Interactions between the Li^+^ cation and the EO3 side chain negatively impacted the transport and physical properties of the electrolyte. We believe the EO1 cation behaved similarly to a noncoordinating solvent and generally improved the properties of the electrolyte. EO3 interacts with the Li^+^ cation through its ether side chain, but the interaction did not lead to desirable improvements in the physical or transport properties of the electrolyte. Modifying the functionality of the IL used in these ternary electrolytes may further inform how IL functionality can be leveraged to tune the properties of highly solvating electrolyte systems.

## Supplementary Material



## Notes

Synthesis methods of ILs, DSC thermograms, oxidative stability of electrolyte compositions; lithium deposition and stripping Coulombic efficiencies; tabulated data values, ^1^H, ^19^F, ^7^Li single-pulse NMR spectra, and tables of ^13^C chemical shift values (PDF)
