# Supplementary material for: Influence of Ether-Functionalized Pyrrolidinium Ionic Liquids on Properties and Li+ Cation Solvation in Solvate Ionic Liquids
Source: J Phys Chem C Nanomater Interfaces. 2025 Jun 10;129(24):10802–14. doi: 10.1021/acs.jpcc.5c01403 (PMC12186621; doi:10.1021/acs.jpcc.5c01403)
Supplement: Supplementary file 1 [file jp5c01403_si_001.pdf]

Supporting Information

Influence of Ether-Functionalized Pyrrolidinium Ionic Liquids on Properties and Li^+ Cation Solvation in Solvate Ionic Liquids

Michael J. Keating[a,b], Elijah Bernard[a,c], Martina Hove[a,c], Ho Martin Yuen[c], Mehreen Mughal[c], Surabh S. KT [a], James F. Wishart[d], Sharon Lall-Ramnarine[c], Robert J. Messinger[a,b], Elizabeth J. Biddinger[a,b]

[a] *Department of Chemical Engineering, The City College of New York, CUNY, New York, NY 10031, USA*

[b] *Ph.D. Program in Chemistry, The Graduate Center of City University of New York, CUNY, New York, NY 10016, USA*

[c] *Department of Chemistry, Queensborough Community College of The City University of New York, Bayside, New York 11364, USA*

[d] *Chemistry Division, Brookhaven National Laboratory, Upton, New York 11973, USA*

[*]e-mail: Elizabeth Biddinger: ebiddinger@ccny.cuny.edu

Materials

All chemicals and reagents were obtained from Sigma Aldrich and used without further purification, except for lithium bis(trifluoromethylsulfonyl)amide, which was obtained from IoLiTec, Inc. and 2-(methoxyethoxyethoxy)ethyl bromide (BrEOEOEOM), which was purchased from TCI America. ^{1}H and ^{13}C NMR spectra were recorded on a Bruker 400 MHz NMR spectrometer.

Synthesis of *N*-(2-methoxyethyl)-*N*-methylpyrrolidinium Bromide (EOMmPyrr Br)

N-Methylpyrrolidine (12.5 mL, 18.0 g, 0.1174 mol) was dissolved in 25 mL acetonitrile in a 3-neck round bottom flask fitted with a stir bar. To the mixture was added dropwise one molar equivalent (12.5 mL, 16.32 g, 0.1174 mol) 2-bromoethylmethyl ether dissolved in 35 mL acetonitrile. The reactants were measured out in a glove tent under dry air and the flask was purged with argon, capped and placed in an ice bath under the hood before the addition started. The flask was fitted with a reflux condenser and the reaction was left to stir at room temperature for six days. The reaction mixture was rotary evaporated under reduced pressure at 40 °C. The product was an orange-colored viscous liquid (27.43 g, 55% yield, $C_8H_{18}NOBr$, Molar mass: 224.17 g/mol). ^{1}H(400 MHz; DMSO-d_6) δ 3.74-3.78(t, 2H), 3.54-3.58 (t, 2H), 3.48-3.49 (t, 4H), 3.31 (s, 3H), 3.02 (s, 3H), 2.08 (s, 4H); ^{13}C(101 MHz; DMSO-d_6) δ 66.00, 64.11, 61.98, 58.13, 48.50, 20.87.

Synthesis of *N*-(2-methoxyethyl)-*N*-methylpyrrolidinium Bis(trifluoromethylsulfonyl)imide (EOMmPyrr TFSI)

EOMmPyrr Bromide (27.43 g, 0.1224 mol) was dissolved in 20 mL water in a round bottom flask fitted with a stir bar. To the mixture lithium bis(trifluoromethylsulfonyl)imide (39.36 g, 0.1370 mol, 10% excess) dissolved in 20 mL water was added slowly. The reaction mixture was left to stir for 24 hours at room temperature. The water layer was then extracted and the product washed with 140 mL (7 x 20mL) of deionized water until the wash tested negative for bromide with 50 mM aqueous silver nitrate. The resulting yellow liquid was rotary evaporated and dried in a high vacuum oven at 50 °C for several days. The final product was a pale yellow liquid (43.75 g, 84% yield, $C_{10}H_{18}S_2N_2O_5BrF_6$), Molar mass: 424.38 g/mol). ^{1}H(400 MHz; DMSO-d_6) δ 3.74-3.76(t, 2H), 3.54-3.58 (t, 2H), 3.48-3.49 (t, 4H), 3.31 (s, 3H), 3.02 (s, 3H), 2.08 (s, 4H); ^{13}C(101 MHz; DMSO-d_6) δ 124.74, 121.05, 117.85, 115.15, 65.97, 64.11, 62.00, 58.10, 48.01, 20.85.

Synthesis of *N*-methyl-*N*-(2-(2-(2-methoxyethoxy)ethoxy)ethyl)pyrrolidinium bromide (EOEOEOMmpyrr bromide)

To *N*-methylpyrrolidine (5.13 g, 6.3 mL, 0.0602 mol) dissolved in 30 mL of acetonitrile in a three-neck round bottom flask fitted with a stir bar, was added dropwise one equivalent of 1-(2-bromoethoxy)-2-(2-methoxyethoxy)ethane (13.67 g, 15.0 mL, 0.0602 mol) dissolved in 20 mL of acetonitrile. The reactants were measured out separately in a glove tent under nitrogen gas and the reaction flask was purged with nitrogen gas while the reactants were being combined. The reaction mixture was set up in an ice bath and left to stir at room temperature for several days. The product was purified by washing with ethyl acetate, then rotary evaporated to remove residual solvent and dried in a high vacuum oven at 50 °C. The resulting compound was a pale yellow liquid (14.00 g, 75 %, $C_{12}H_{26}NO_3Br$, Molar mass: 312.3 g/mol). ^{1}H(400 MHz; DMSO-d_6) δ 3.85 (t, 2H), 3.54-3.57

(m, 12H), 3.43-3.45 (m, 2H), 3.25 (s, 3H), 3.04 (s, 3H), 2.08 (d, 4H); ^{13}C(101 MHz; D_2O) δ 71.24, 69.44, 69.42, 69.37, 64.44, 64.14, 62.03, 58.06, 47.86, 20.82.

Synthesis of *N*-methyl-N-(2-(2-(2-methoxyethoxy)ethoxy)ethyl)pyrrolidinium bis(trifluoromethylsulfonyl)imide (EOEOEOMmPyrr TFSI)

EOEOEOMmPyrr bromide (14.00 g, 0.0448 mol) was reacted with lithium bis(trifluoromethylsulfonyl)amide (14.19 g, 0.0494 mol, 10% excess) dissolved in 50 mL of distilled water. The reaction mixture was left to stir at room temperature for 24 hours. The resulting liquid was washed with distilled water and methylene chloride until the wash tested negative for bromide with 50 mM aqueous silver nitrate. The product was then rotary evaporated and dried in a high vacuum oven at 60 °C for several days. The final product was a pale yellow liquid (15.69 g, 68 %, $C_{14}H_{26}S_2N_2O_7F_6$, Molar mass 512.5 g/mol). ^{1}H(400 MHz; DMSO-d_6) δ 3.85 (t, 2H), 3.54-3.57 (m, 12H), 3.43-3.45 (m, 2H), 3.25 (s, 3H), 3.04 (s, 3H), 2.08 (d, 4H); ^{13}C(101 MHz; DMSO-d_6) δ 124.40, 121.20, 118.00, 114.80, 71.24, 69.44, 69.42, 69.37, 64.44, 64.14, 62.03, 58.06, 47.86, 20.82.

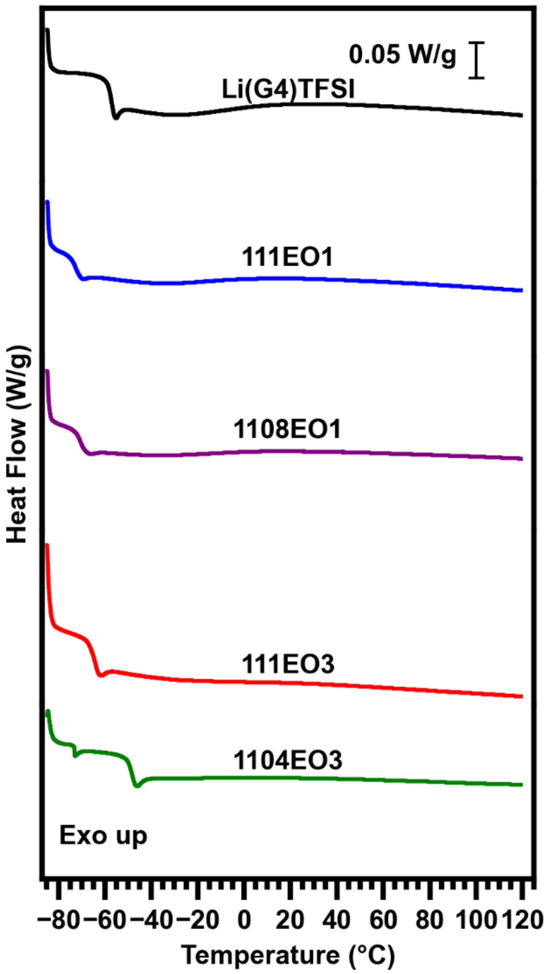

Figure S1: DSC thermograms of heating from -85 °C to 120 °C

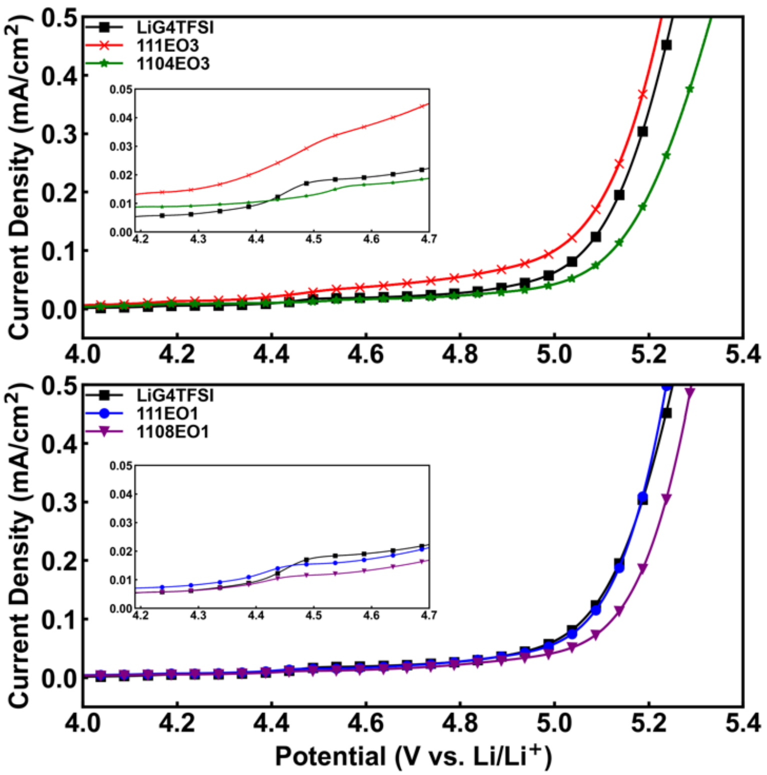

Figure S2: Linear sweep voltammetry with inset of ether oxidation

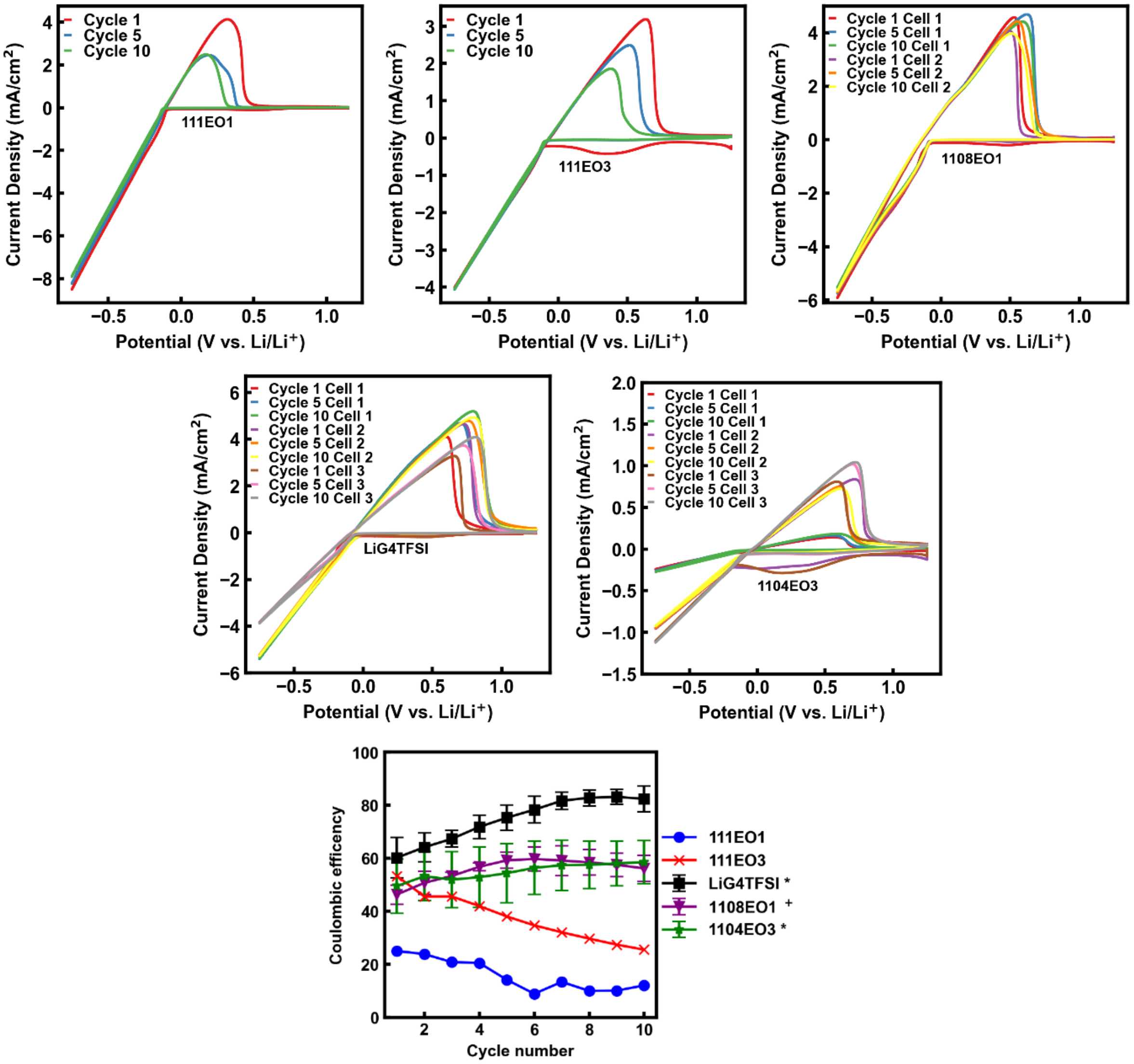

Figure S3. Cyclic voltammograms of LiG4TFSI and ternary compositions and coulombic effiecincies from cyclic voltametry at 30 °C through 10 cycles of ternary composition. Error bars represent standard deviation,* average of 3 cells, [+] average of 2 cells

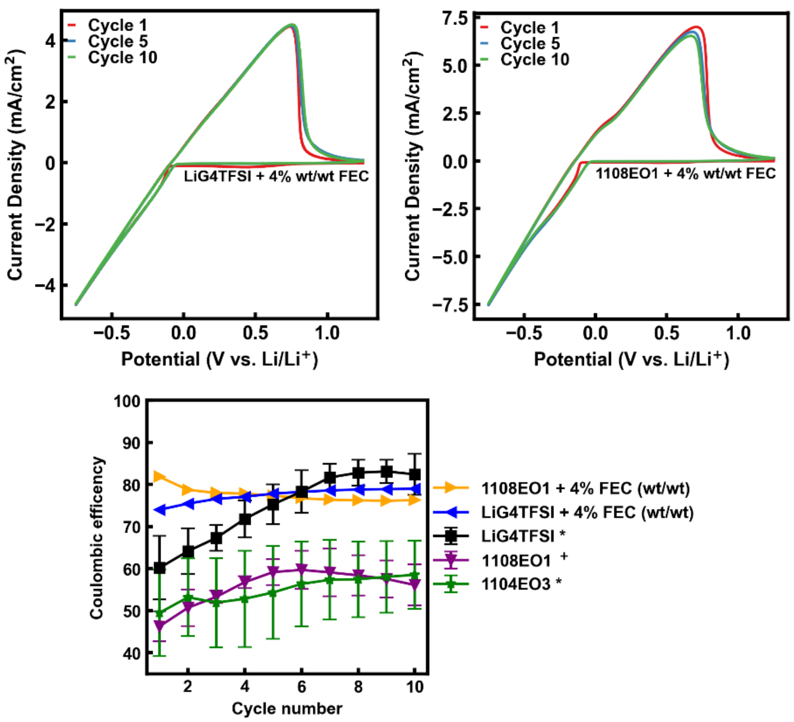

Figure S4: Cyclic voltammograms with FEC additive and coulombic effiecincies at 30 °C through 10 cycles of ternary samples with [O][Li$^+$]=5, with and without 4% FEC additive. Error bars represent standard deviation,* average of 3 cells, $^+$ average of 2 cells

Table S1. Numerical data for conductivity (σ, mS/cm)

°C	LiG4TFSI	111EO1	1108EO1	111EO3	1104EO3
**-40**	7.86E-05	2.71E-03	1.14E-03	2.55E-04	1.15E-06
**-30**	1.58E-03	0.0171	8.02E-03	2.66E-03	4.82E-05
**-20**	0.0135	0.0712	0.0364	0.0158	5.91E-4
**-10**	0.0635	0.212	0.119	0.0622	3.94E-3
**0**	0.196	0.493	0.299	0.179	0.0149
**10**	0.467	0.959	0.623	0.410	0.0515
**20**	0.906	1.64	1.133	0.804	0.131
**30**	1.53	2.54	1.85	1.37	0.279
**40**	2.35	3.68	2.80	2.15	0.517
**50**	3.38	5.06	4.00	3.13	0.891
**60**	4.59	6.66	5.40	4.32	1.40

Table S2. Numerical data for viscosity (η, cP)

°C	LiG4TFSI	111EO1	1108EO1	111EO3	1104EO3
**0**	668.2	314	594.32	550.4	11239.6
**5**	423.4	223.6	397.83	361.7	6502.4
**10**	288.4	164.7	282.85	257.5	3595.4
**15**	201.2	122.4	203.61	182.4	2311.4
**20**	148.3	95	153.8	137	1451
**25**	110.2	73.4	116.44	102.3	989.3
**30**	88.7	59.3	93.81	83.1	506.4
**35**	69.1	48.3	73.2	64.9	356.7
**40**	55.4	38.9	59.68	52.4	266
**45**	44.5	32.1	48	42	201.5
**50**	36.6	27	39.79	34.6	157.2
**55**	30	23.3	32.52	28.6	122.3
**60**	25.7	20	27.66	24.5	97.7
**65**	21.9	17.4	23.67	21	79.9
**70**	19	15.2	20.45	18.3	63.6
**75**	16.6	13.5	17.98	16	53.2
**80**	14.6	12	15.73	14.1	43.7
**85**	12.9	10.7	13.95	12.4	37.1
**90**	11.4	9.5	12.42	11.1	30.9
**95**	10.2	8.5	11.1	10	26.9

Table S3. Numerical data for density (ρ, g/cm^3)

°C	LiG4TFSI	111EO1	1108EO1	111EO3	1104EO3

**10**	1.4158	1.434	1.4592	1.4102	1.4952
**20**	1.4048	1.4236	1.4481	1.4001	1.4848
**25**	1.3995	1.4185	1.4409	1.3950	1.4797
**30**	1.3943	1.4134	1.4352	1.3901	1.4746
**40**	1.3840	1.4033	1.4243	1.3803	1.4646
**50**	1.3739	1.3934	1.4139	1.3706	1.4547
**60**	1.3640	1.3836	1.4043	1.3606	1.4449
**70**	1.3542	1.374	1.3952	1.3515	1.4353

$$MW_{Composition} = \chi_{SIL}(MR_{G4}MW_{G4} + MW_{LiTFSI}) + \chi_{IL}MW_{IL} \qquad Eq\ S1$$

$$M_{Composition} = \rho_{Composition} — MW_{Composition} \qquad Eq\ S2$$

$$\Lambda_{imp} = \sigma_{Composition} — M_{Composition} \qquad Eq\ S3$$

Table S4: Table of data from figure 2 and figure 3 including the calculated molarity (M), calculated Λ_{imp}, diffusion ratios and the ionicity ($\Lambda_{imp}/\Lambda_{NMR}$), all data represented at 30°C

Name	η (cP)	σ (mS cm^{-1})	ρ (g cm^{-3})	M (mol dm^{-3})	Λ_{imp} (mS cm^2 mol^{-1})	D_{G4}/D_{Li}	D_{cat}/D_{Li}	$\Lambda_{imp}/\Lambda_{NMR}$	Walden Product (P S cm^2 mol^{-1})
**LiG4TFSI**	88.7	1.53	1.394	2.74	558.1	0.991	N/A	0.69	0.495
**111EO1**	59.3	2.54	1.413	3.03	837.5	0.981	1.253	0.71	0.496
**1108EO1**	93.8	1.85	1.435	3.23	572.6	1.099	1.481	0.72	0.537
**111EO3**	83.1	1.37	1.390	2.72	503.5	1.040	0.971	0.67	0.418
**1104EO3**	506.4	0.28	1.475	3.32	83.9	1.241	1.019	0.64	0.424

Table S5. Table of Raman fitting area ratio and peak positions

Name	$Area_{CIP}$ / $Area_{SSIP}$	Peak position CIP	Peak position SSIP
**LiG4TFSI**	0.255	746.32	741.04
**111EO1**	0.105	746.73	741.12

**1108EO1**	0.323	747.34	741.66
**111EO3**	0.132	746.41	740.95
**1104EO3**	0.271	747.35	741.44
**LiTFSI**		747.15	

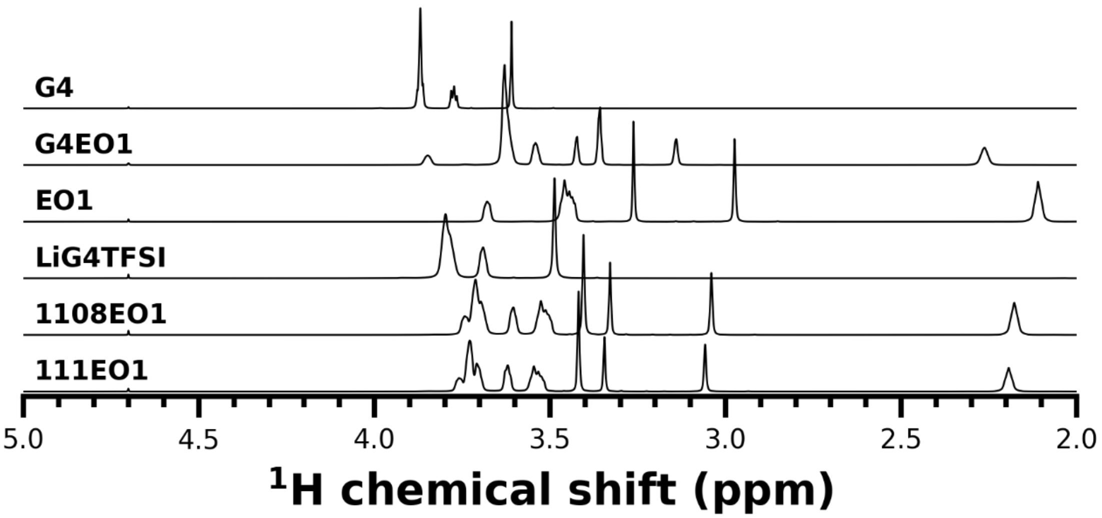

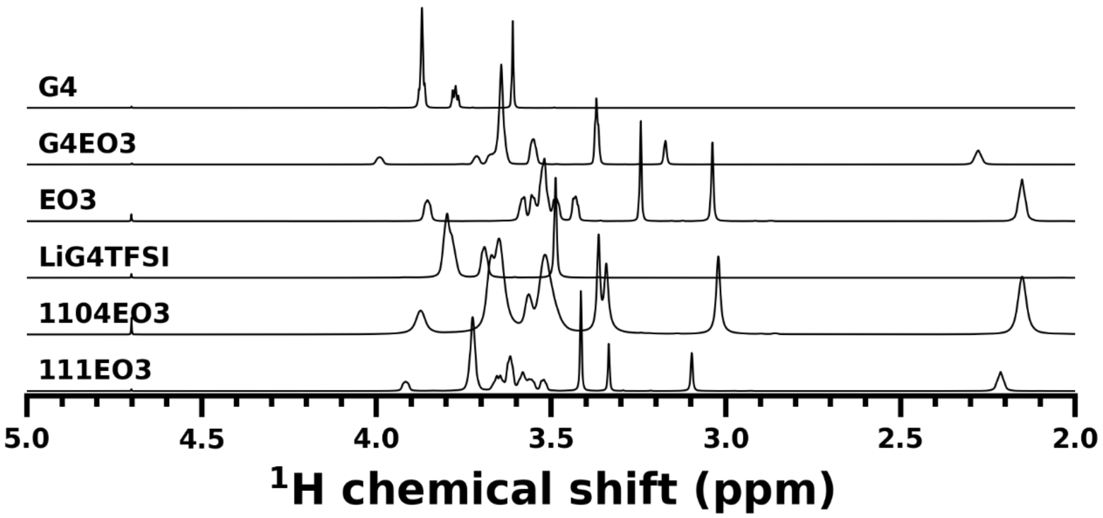

Figure S5. Liquid-state ^{1}H single-pulse NMR of LiG4TFSI and ternary electrolyte composition.

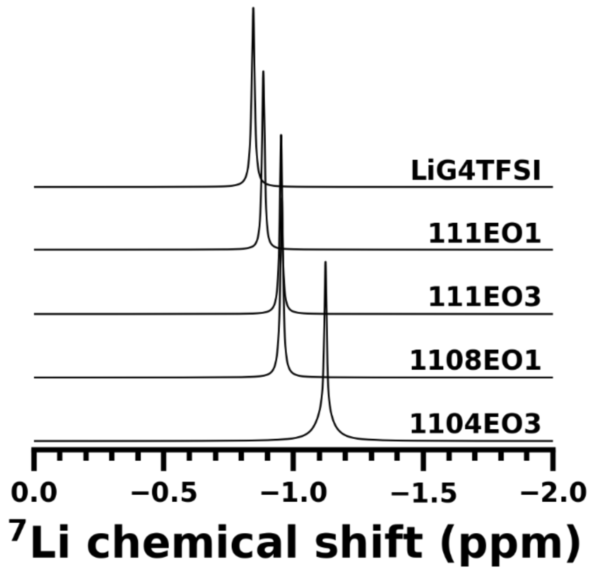

Figure S6. Liquid-state ^{7}Li single-pulse NMR of LiG4TFSI and ternary electrolyte composition

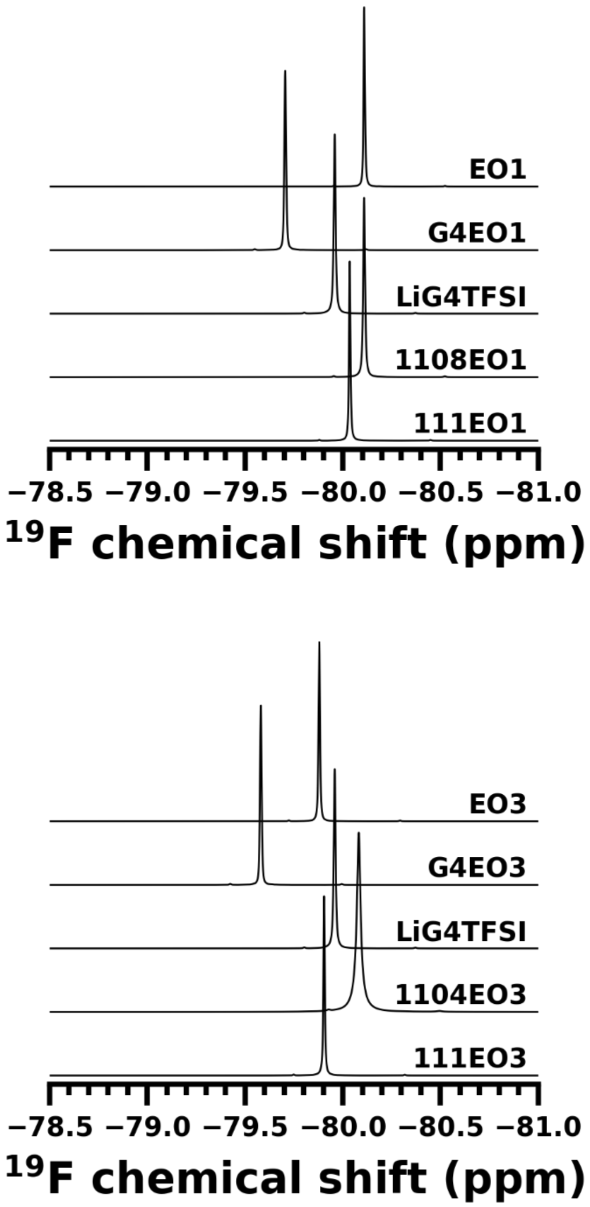

Figure S7. Liquid-state ^{19}F single-pulse NMR of LiG4TFSI and ternary electrolyte composition

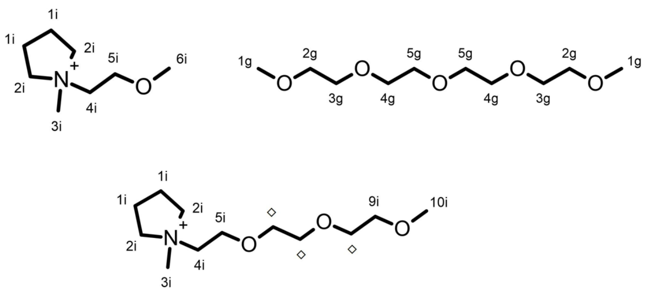

Figure S8. NMR carbon positional assignments

Table S6. Liquid-state ^{13}C single-pulse NMR peak positions values in ppm for EO1 composition. Assignments from Figure S8

**LABEL**	**EO1**	**G4**	**LiG4TFSI**	**G4EO1**	**111EO1**	**1108EO1**
1g		58.508	58.372	57.924	58.281	58.251
2g		72.237	70.032	71.769	69.888	69.831
3g		70.666	67.686	70.034	67.503	67.569
4g		70.822	67.963	70.243	67.808	67.867
5g		70.842	68.367	70.252	68.204	68.159
1i	20.756			21.064	20.839	20.813
2i	64.975			65.083	65.010	65.026
3i	48.187			48.440	48.242	48.237
4i	62.940			63.123	63.020	63.012
5i	65.958			66.298	66.081	66.044
6i	57.883			58.168	57.988	57.956

Table S7. Liquid-state ^{13}C single-pulse NMR peak positions values in ppm for EO3 composition. Assignments from Figure S8

LABEL	EO3	G4	LiG4TFSI	G4EO3	111EO3	1104EO3
1g		58.508	58.372	58.003	58.279	58.213
2g		72.237	70.032	71.809	70.188	69.825
3g		70.666	67.686	70.078	67.945	67.158
4g		70.822	67.963	70.283	68.159	67.808
5g		70.842	68.367	70.283	68.551	68.157
1i	20.883			21.117	20.911	20.779
2i	65.134			65.212	65.139	64.995
3i	48.163			48.355	48.196	48.113
4i	64.685			64.950	64.753	64.594
5i	69.810			70.063	69.616	69.328
6i	◊			◊	◊	◊
7i	◊			◊	◊	◊
8i	◊			◊	◊	◊
9i	71.518			71.766	71.323	70.389
10i	57.758			58.027	57.988	58.177
